# Data set for phylogenetic tree and RAMPAGE Ramachandran plot analysis of SODs in *Gossypium raimondii* and *G. arboreum*

**DOI:** 10.1016/j.dib.2016.05.025

**Published:** 2016-06-18

**Authors:** Wei Wang, Minxuan Xia, Jie Chen, Fenni Deng, Rui Yuan, Xiaopei Zhang, Fafu Shen

**Affiliations:** State Key Laboratory of Crop Biology, College of Agronomy, Shandong Agricultural University, Tai’an 271018, Shandong, PR China

**Keywords:** Phylogenetic tree, RAMPAGE Ramachandran plot analysis, SOD, Cotton

## Abstract

The data presented in this paper is supporting the research article “Genome-Wide Analysis of Superoxide Dismutase Gene Family in *Gossypium raimondii* and *G. arboreum*” [1]. In this data article, we present phylogenetic tree showing dichotomy with two different clusters of SODs inferred by the Bayesian method of MrBayes (version 3.2.4), “Bayesian phylogenetic inference under mixed models” [2], Ramachandran plots of *G. raimondii* and *G. arboreum* SODs, the protein sequence used to generate 3D sructure of proteins and the template accession via SWISS-MODEL server, “SWISS-MODEL: modelling protein tertiary and quaternary structure using evolutionary information.” [3] and motif sequences of SODs identified by InterProScan (version 4.8) with the Pfam database, “Pfam: the protein families database” [4].

**Specifications Table**

**Table:** 

Subject area	Biology
More specific subject area	Genetics and Molecular Biology
Type of data	Figure
How data was acquired	Database analysis
Data format	Analyzed
Experimental factors	Amino acid sequences were retrieved from NCBI, TAIR10, Joint Genome Institute (JGI) and/or CottonGen.
Experimental features	Sequences were aligned using BLAST for Proteins (BLASTP), Structural evaluation and stereochemical analyses were assessed using RAMPAGE Ramachandran plot analysis
Data accessibility	With this article

**Value of the data**

• Data on phylogenies separately estimated using Bayesian method of MrBayes enable researchers to examine how the topologies differ from each other.
• Data on phylogenies of Gossypium SOD proteins enable researchers to infer the possible ranges of time frames in the divergence events of Gossypium *SOD* genes and its molecular evolution in general.
• Data on RAMPAGE Ramachandran plot analysis of Gossypium SOD proteins enable researchers to evaluate the accuracy of the predicted models.

## 1 Data

The phylogenetic tree obtained using Maximum-Likelihood (ML) method of PhyML (version 20120412) [5] and the 3D structure of SODs generated by using SWISS-MODEL server (http://swissmodel.expasy.org/) [3] and using the online COACH server (http://zhanglab.ccmb.med.umich.edu/COACH/) [6]. were presented in [1]. The data shown here represent the showing dichotomy with two different clusters of SODs (I: Cu/Zn; II: Mn/Fe-SODs) inferred by the Bayesian method of MrBayes (version 3.2.4) [2] and Cu/Zn-SOD cluster had three subgroups (Ia–Ic), whereas the Mn/Fe-SOD cluster had two subgroups (IId and IIe) (Figure. 1). We analysed the accuracy of the predicted models evaluated by Ramachandran plot using the RAMPAGE server (http://mordred.bioc.cam.ac.uk/~rapper/rampage.php) [3]. The refined SOD models showed good proportions of residues in favoured, allowed and outlier regions (Appendix A, Appendix A). In-depth analyses of the data is presented in the associated research article [1].

## 2 Experimental design, materials and methods

### 2.1 Information access

The latest versions of the *G. raimondii* (V1.0) and *G. arboreum* (V2.0) genomes and annotation files were downloaded from CottonGen (https://www.cottongen.org/data/genome). The latest version of the Arabidopsis (TAIR10) genome and annotation files were downloaded from the Joint Genome Institute (JGI) (http://www.phytozome.net).

### 2.2 Data filtering

We then filtered gene annotation results based on the following criteria [7]: (1) the longest transcript in each gene loci was chosen to represent that locus; (2) coding sequences (CDS) with length <150 base pair bp were filtered out; (3) CDS with the percentage of ambiguous nucleotides (‘N’) >50% were filtered out; (4) CDS with internal termination codon were filtered out; and (5) the CDS with hits(Basic Local Alignment Search Tool (BLAST) identity ≥80%) to RepBase sequences (http://www.girinst.org/repbase/index.html) were filtered out.

### 2.3 Identification of SOD protein

To identify members of the SOD protein in *G. raimondii* and *G. arboreum*, we retrieved SOD protein sequences from the NCBI protein database (http://www.ncbi.nlm.nih.gov/protein/). These protein sequences from six species, including Arabidopsis (accession nos. NP_172360.1, NP_565666.1, NP_197311.1, NP_199923.1, NP_197722.1 and NP_187703.1), Theobroma cacao (XP_007030135.1 and XP_007038205.1), G. hirsutum (ABA00453.1, ACC93639.1, ABA00454.1, ABA00456.1 and ABA00455.1), Po. trichocarpa (XP_002319589.1 and XP_002325843.1), Z. mays (NP_001105704.1, BAI50563.1, ACG41865.1, ACG32380.1 and NP_001105742.1) and O. sativa (AAA33917.1, BAD09607.1, BAA37131.1 and NP_001055195.1), were used as query sequences to perform multiple database searches using BLAST for Proteins (BLASTP) [8]. After removing alignments with identity <50%, the resultant candidate SOD proteins were aligned to each other to ensure that no gene was represented multiple times. InterProScan (version 4.8) [9]was further used to confirm the inclusion of the SOD domain in each candidate sequence using the Pfam database. Furthermore, we gathered the SOD protein sequences, the template accession and motif sequences.

### 2.4 Construct phylogenetic trees

Phylogenetic trees were constructed using the Bayesian analysis method. Bayesian trees were constructed using MrBayes (version 3.2.4) [2] with GTR+I+gamma substitution model. The Markov chain Monte Carlo process performed 5,000,000 iterations with sampling every 500 iterations resulting in 10,000 samples and a burn-in of 25% samples. Other parameters were the default settings.

### 2.5 Structural evaluation and stereochemical analysis

Structural evaluation and stereochemical analyses were assessed using RAMPAGE Ramachandran plot analysis (http://mordred.bioc.cam.ac.uk/~rapper/rampage.php) [10].

## Footnotes

Appendix A Supplementary data associated with this article can be found in the online version at doi:10.1016/j.dib.2016.05.025.
