# Supplementary material for: Data set for phylogenetic tree and RAMPAGE Ramachandran plot analysis of SODs in Gossypium raimondii and G. arboreum
Source: Data Brief. 2016 Jun 18;9:345–8. doi: 10.1016/j.dib.2016.05.025 (PMC5030311; doi:10.1016/j.dib.2016.05.025)
Supplement: Supplementary file 1 — Supplementary material [file mmc1.doc]

**Conflict of interest**

The authors declared that they have no conflicts of interest to this work. We declare that we do not have any commercial or associative interest that represents a conflict of interest in connection with the work submitted.
