# Supplementary figures and images for: Data set for phylogenetic tree and RAMPAGE Ramachandran plot analysis of SODs in Gossypium raimondii and G. arboreum
Source: Data Brief. 2016 Jun 18;9:345–8. doi: 10.1016/j.dib.2016.05.025 (PMC5030311; doi:10.1016/j.dib.2016.05.025)

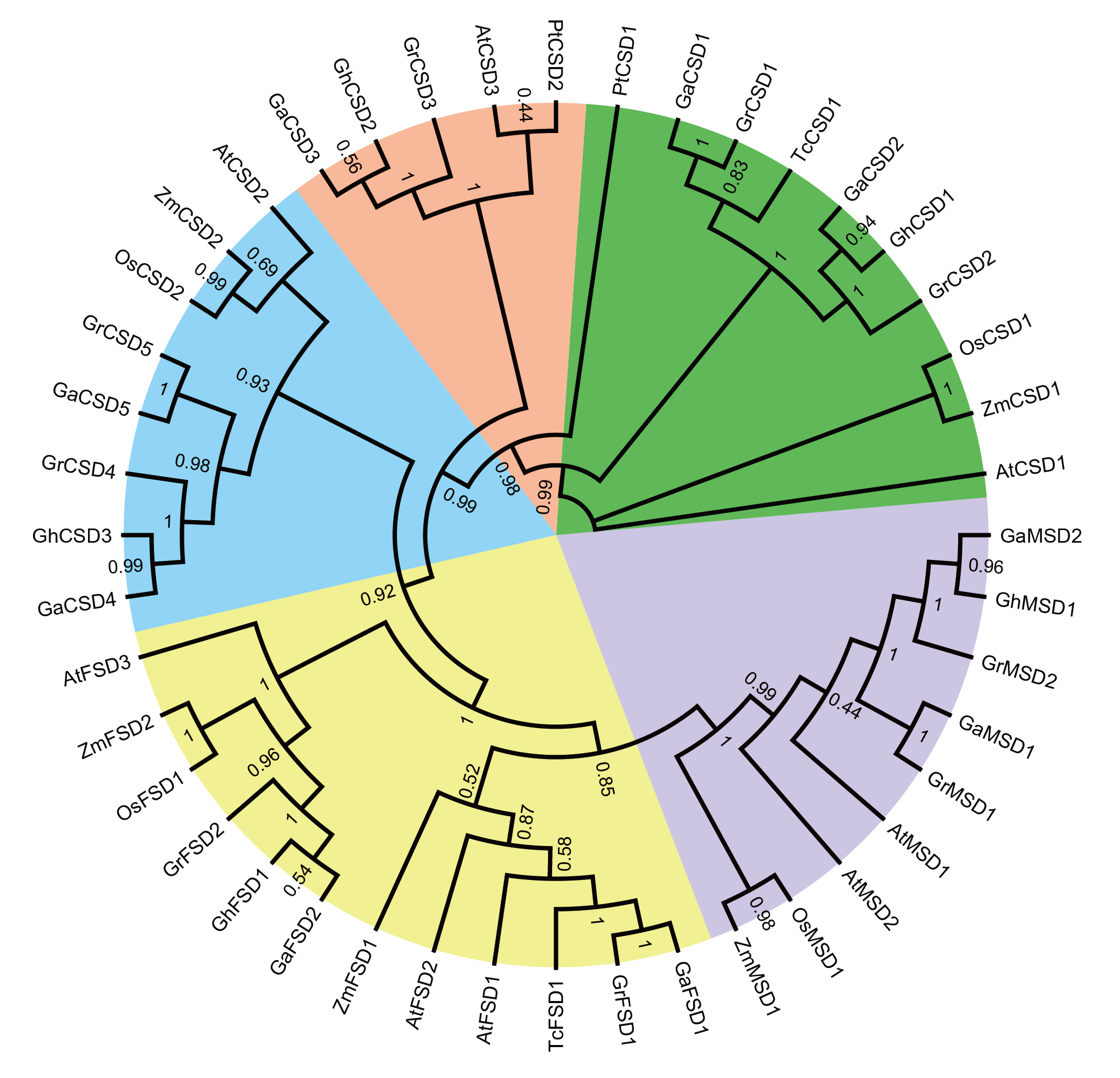

Supplement: Supplementary file 2 — Supplementary material [file mmc2.zip › Figure S1.png]

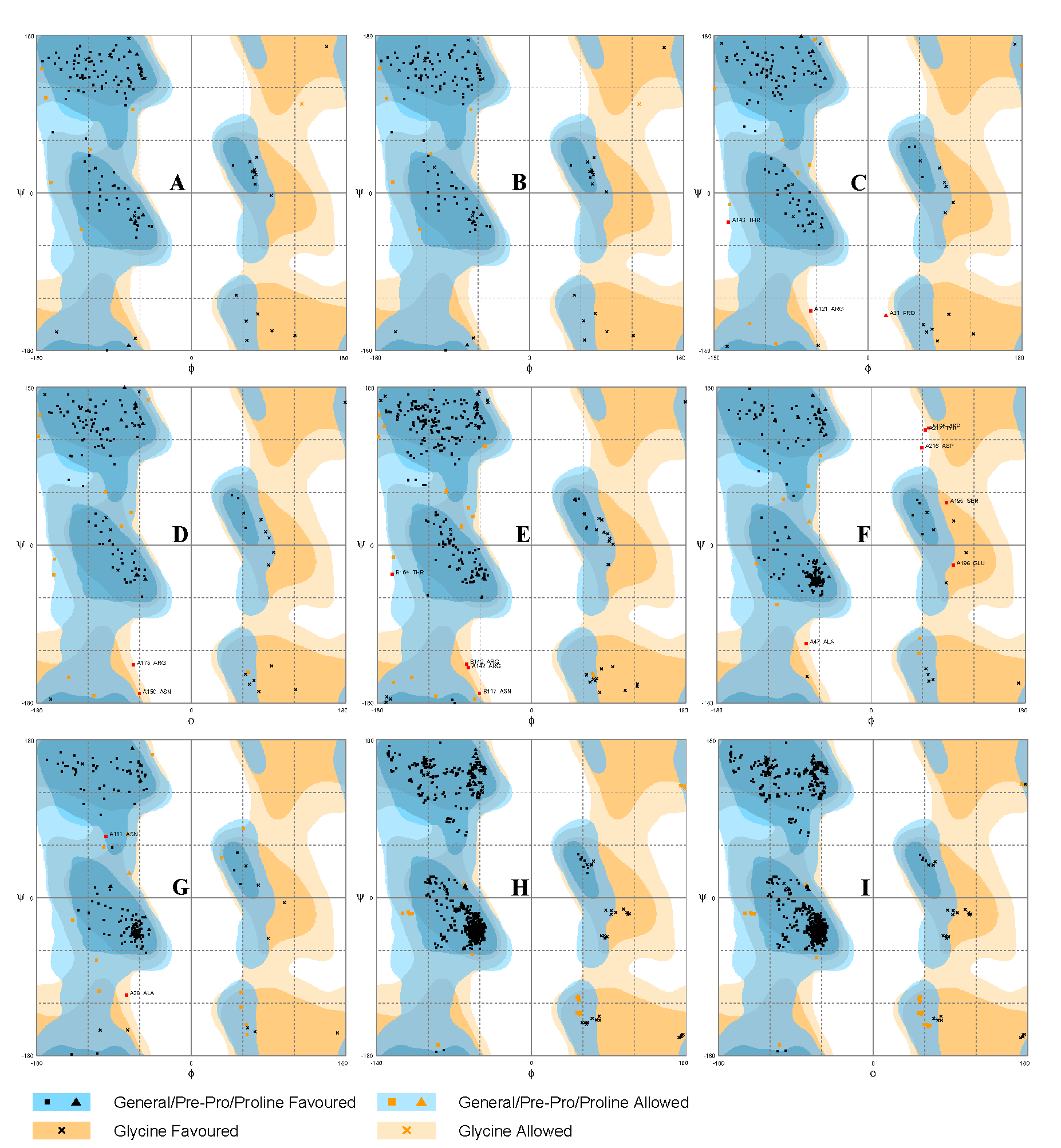

Supplement: Supplementary file 3 — Supplementary material [file mmc3.zip › Figure S2. Ramachandran plots of G. raimondii SODs.png]

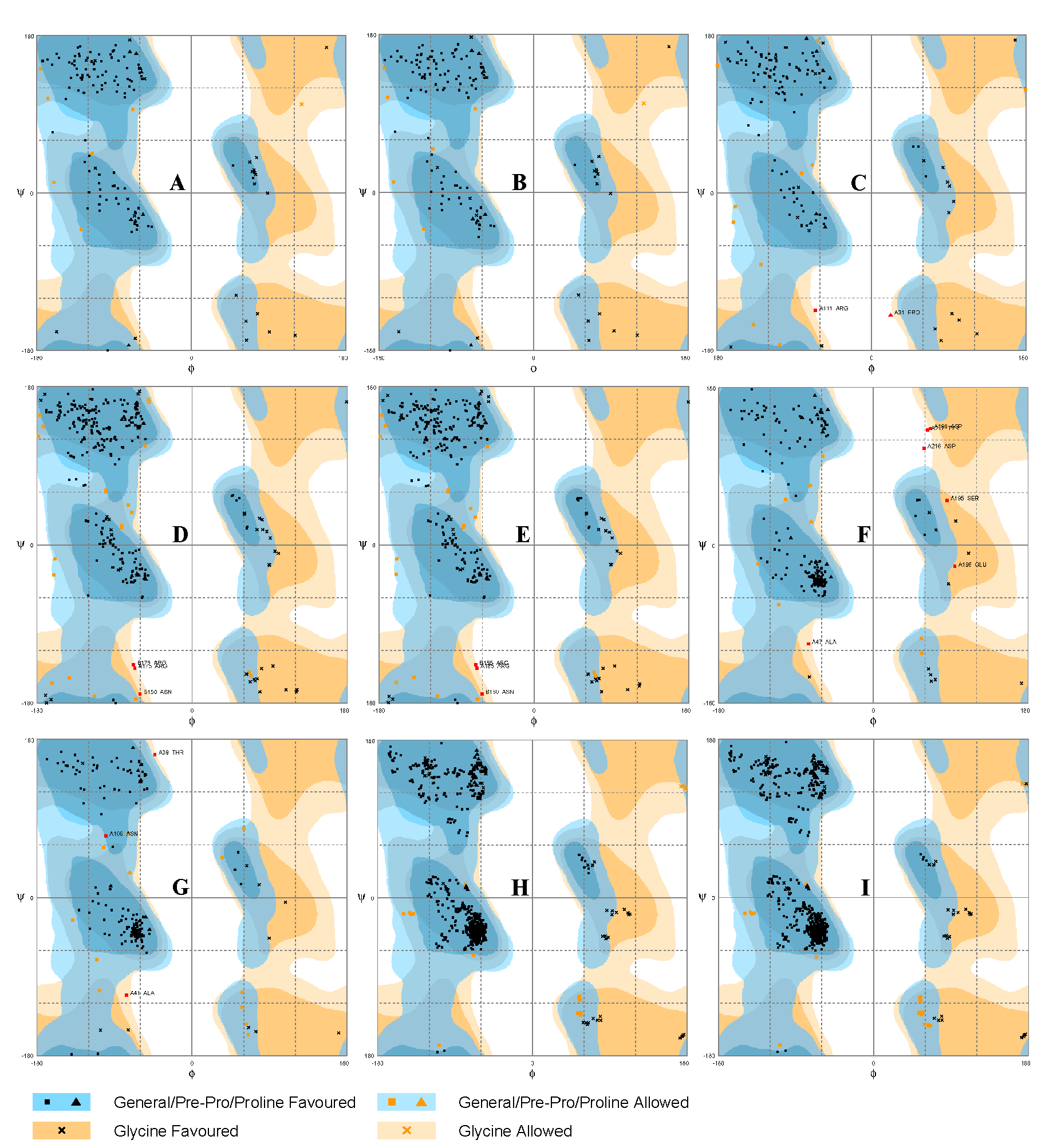

Supplement: Supplementary file 4 — Supplementary material [file mmc4.zip › Figure S3 Ramachandran plots of G. arboretum SODs.png]
